# Detecting *Clostridium botulinum*

**DOI:** 10.3201/eid1208.051364

**Published:** 2006-08

**Authors:** Josef Karner, Franz Allerberger

**Affiliations:** * Medical University Innsbruck, Innsbruck, Austria

**Keywords:** biological warfare, Clostridium botulinum, letter

To the Editor: In the October 2005 issue of Emerging Infectious Diseases, Song et al. described a fiber-optic, microsphere-based, high-density array composed of 18 species-specific probe microsensors, used to identify biological warfare agents, including Clostridium botulinum (*1*). Although the researchers used multiple probes for C. botulinum, we doubt that this approach is suitable for this organism.

C. botulinum comprises a heterogenous group of subspecies that produce botulinum neurotoxin (BoNT); identification and characterization usually rely on animal testing that focuses on antigenetically distinct toxins (*2*). Although strains of C. botulinum that do not produce toxins are sometimes isolated from wound infections not related to botulism, some strains of C. butyricum and C. baratii are also able to produce BoNTs.

The mouse bioassay is currently the accepted method for detecting BoNT. In this assay, mice that receive an intraperitoneal injection containing a sample with more than a minimum lethal dose show symptoms of botulinum intoxication and die. ELISAs, which recognize protein antigenic sites, are still less sensitive than the mouse bioassay (*3*).

Because the mouse bioassay requires euthanizing many animals, and results are not available for several hours, new diagnostic methods are needed. For C. botulinum, an organism widely dispersed in the environment, DNA-based methods may not provide the ultimate solution. Rapid methods to detect and differentiate active BoNTs, such as the rapid, mass spectrometry-based, functional method, are promising candidates to substitute for animal testing in the near future (*4*).
